# Variations in Aggressive and Indolent Behaviour of Central Dentinogenic Ghost Cell Tumor

**DOI:** 10.1155/2020/8837507

**Published:** 2020-11-10

**Authors:** Radhika Manoj Bavle, Sudhakara Muniswamappa, Soumya Makarla, Reshma Venugopal

**Affiliations:** Department of Oral and Maxillofacial Pathology, Krishnadevaraya College of Dental Sciences and Hospital, Bangalore 562157, India

## Abstract

Among the aggressive odontogenic tumors, a few tumors generally ameloblastomas, carry a connotation of being aggressive. But, a rare tumor like central dentinogenic ghost cell tumor (DGCT) can be equally aggressive with a propensity for recurrence. The two cases discussed in this article are divergent in behaviour with features such as presentation, seen in early decades as central intraosseous tumors in the maxilla and mandible. The first case describes an aggressive DGCT, associated with impacted tooth and odontome which showed recurrence into a large solid tumor within a year. The second case arose in accordance with a partially erupted molar which was comparatively innocuous and less aggressive. Both the cases exhibited classic histopathological features. These two unconventional cases of DGCT with a follow-up are being reported here to highlight the variation in behaviour and presentation and also to understand the aggressive nature of the tumor.

## 1. Introduction

In the category of odontogenic tumors, ameloblastomas tend to display a great variety of histopathological patterns, but some lesions like calcifying odontogenic cysts (COC) have shown an intriguing evolution pattern in tumor pathology in the passing decades.

Gorlin et al. first identified and described COC as an entity of odontogenic origin [1]. He elucidated the histomorphological cellular features displayed by COC, both cystic and tumor counterparts [2]. Reporting of more cases with variations led to contemplate a dualistic concept with emergence of names like dentinogenic ghost cell tumor (DGCT) with cystic and solid patterns [1].

Praetorius et al. gave further clarity to the dualistic nature by classifying COC into tumor/solid and cystic lesions with further subclassification as simple, associated with odontome, and associated with ameloblastic proliferation [3]. The World Health Organization (WHO) in its 2005 classification grouped the solid variants separately which led to the emergence of terms calcifying cystic odontogenic tumor (CCOT) and dentinogenic ghost cell tumor (DGCT) representing the cystic and solid neoplastic type, respectively [2, 4, 5]. The recent WHO classification (2017) of odontogenic tumors retains the term DGCT for the solid variant [6, 7].

Here, we present two cases of DGCT. The first case of DGCT seen in a 28-year old male was associated with an impacted canine and odontome; albeit an independent identity as DGCT, it presented as a cyst clinically and was reported as a cystic variety of DGCT. The lesion evolved into a completely solid neoplastic variant of DGCT on recurrence, in other words, type 3 according to Toida et al.'s classification [3]. The other case was in a 21-year old female which was an accidental finding in the lower left molar area in association with a submerged tooth.

### 1.1. Case 1

**Figure 1:**
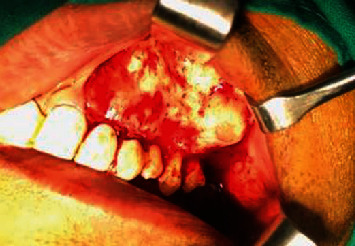
Intraoperative image of case 1 presenting with a mild swelling in the left premolar molar region. The anterior teeth have been root canal treated and restored.

A 28-year old male patient reported to the clinic with a hard maxillary swelling in the left posterior quadrant. On clinical examination, the swelling was seen associated with the premolars and first molar in the left maxillary quadrant (Figure 1). The swelling was bony hard in consistency and was approximately 2 cm in size, with normal stretched mucosa covering it on the palatal and vestibular areas. The examination of teeth revealed a missing canine. The premolars and molars were malaligned but did not show any mobility.

**Figure 2:**
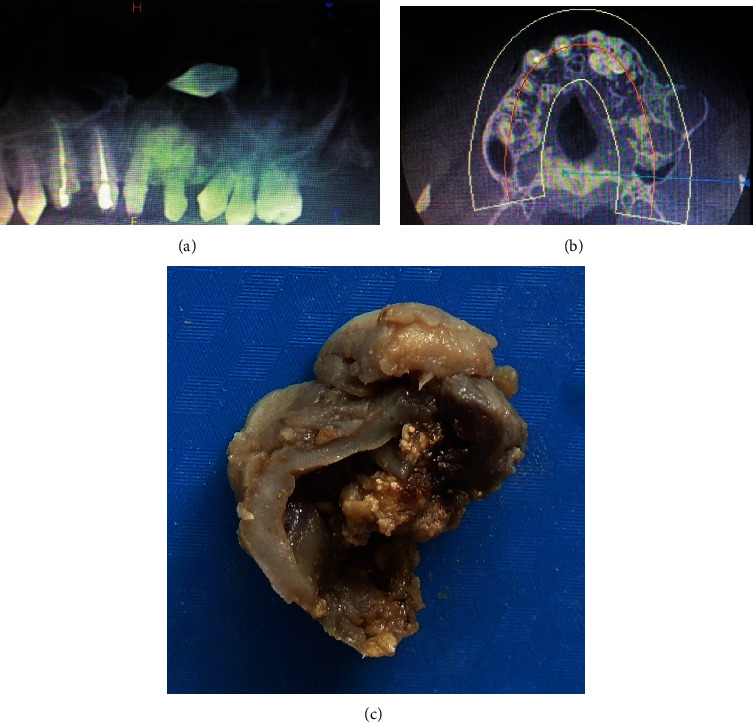
(a) OPG showing impacted canine and radioopaque odontome-like lesion in 23 and 24 regions. Large well-demarcated multilocular lesion irt 24, 25, and 26 regions associated with root resorption. (b) Axial CT scan shows multilocular lesion in the maxillary posterior quadrant with expansion of buccal plate and radio dense lesion irt 23 region. (c) Grossing shows a thick cystic wall with a thick capsule. In the cystic lumen, white gritty areas were observed.

On examination with an orthopantomogram, a fully developed impacted canine was noted in the left antral floor area (Figure 2(a)). In the region of 21 and 22, a radiopaque mass was seen approximately 1.5 cm in diameter. Posterior to the radiopaque mass, a multilocular radiolucency was seen. A computed tomography (CT) scan revealed an impacted canine in association with a radiopaque mass. It extended posteriorly into a large well-defined multilocular radiolucent lesion with specks of radiopacity (Figure 2(b)). The swelling had caused palatal expansion and a notable expansion of the buccal aspect of the alveolar bone. Teeth 24 to 26 were involved in the lesion and showed considerable root resorption. Resorption of the lateral incisor was seen till the mid-half of the root extending into the odontome. The premolar and molar teeth showed resorption of roots till the middle 3^rd^ of the root (Figure 2(a)).

A provisional diagnosis of COC with an impacted canine and odontome was made for the first case and was treated with conservative surgical removal of 23, odontome, and the radiolucent lesion. The radiopaque and radiolucent lesional tissues were removed in toto and appeared to be well circumscribed. Gross examination revealed a soft cystic lesional mass along with maxillary canine (Figure 2(c)).

**Figure 3:**
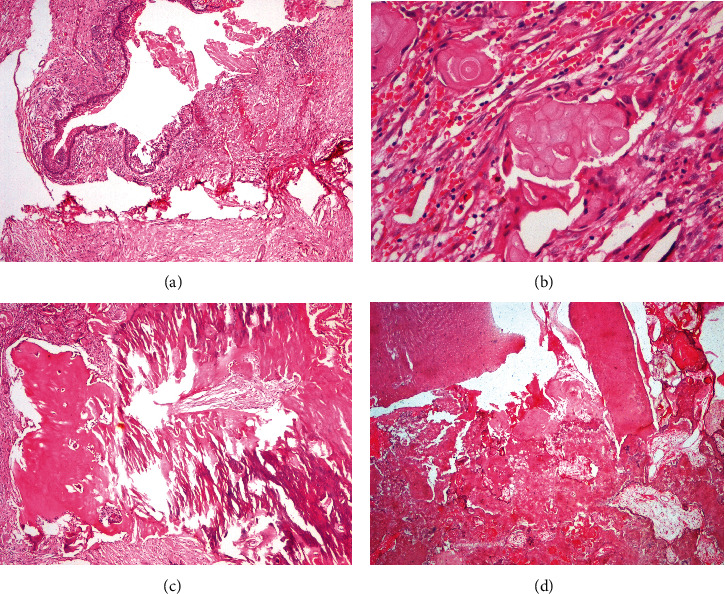
(a) Thick cystic tumor wall, showing loose mature connective tissue. Cystic epithelium is of variable thickness ranging from 10 to 15 cells. The basal cells exhibit reversal of polarity and superficial cells show spongiosis (H&E stain, ×40). (b) Ghost cells are seen in small and large clusters in the connective tissue along with epithelial cells (H&E stain, ×100). (c) Large amount of dentinoid induction with entrapped ghost cells and calcified ghost cells (H&E stain, ×40). (d) Decalcified section showing dentin, cementum-like tissue, and ectomesenchymal tissue arranged haphazardly with entrapped ghost cells (H&E stain, ×40).

On histopathological examination, the hematoxylin and eosin- (H&E-) stained sections showed odontogenic cystic epithelium with tall columnar basal cells with polarized hyperchromatic nuclei. Suprabasal cells resembling the stellate reticulum-like cells and ghost cells were evident (Figure 3(a)). The connective tissue showed odontogenic epithelial islands, nests made of cuboidal cells with multiple areas of ghost cells (Figure 3(b)). The ghost cells showed calcification in a few sites. Varying amounts of mineralized dysplastic dentin and dentinoid were seen in association with odontogenic epithelial cells (Figure 3(c)). Reactive and residual bone was seen at the periphery of the lesion.

The decalcified H&E-stained sections of the hard mass showed dentin, cementum, and ectomesenchymal tissues similar to the dental papilla. All these tissues were arranged haphazardly representing a composite complex odontome (Figure 3(d)). A diagnosis of DGCT with odontome and impacted canine was given.

Follow-up led to the disclosure of recurrence in the maxillary antral floor in the form of a solid tumor (DGCT) after 11 months, but the patient was asymptomatic.

**Figure 4:**
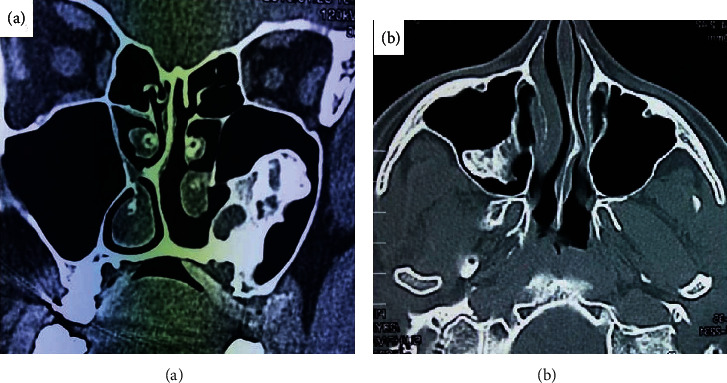
(a, b) CT scan showing a large mass in the left antrum, resting/supported on the floor of the antrum. No distinction of the medial antral wall was seen.

A CT scan was done revealing a lowering mass in the antrum showing radiolucent radiopacities (Figure 4), and a provisional diagnosis of recurrent DCGT was made. The recurrent tumor was treated with surgical therapy. The patient has responded well with no history of recurrence for the last 2 years.

**Figure 5:**
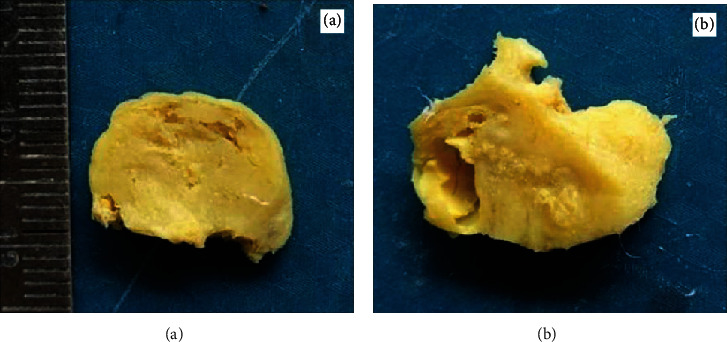
(a, b) Grossing image shows solid mature tissue areas.

**Figure 6:**
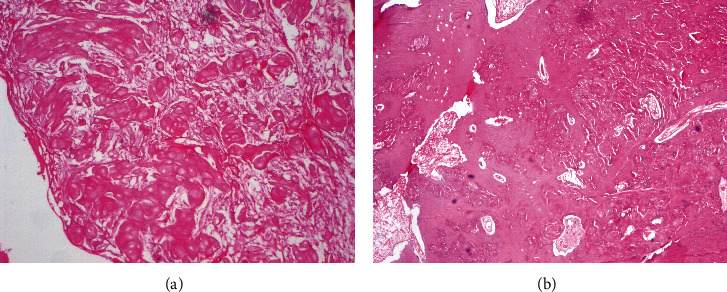
(a) A large number of nests and islands of ghost cells in the connective tissue stroma (H&E stain, ×40). (b) Ghost cells seen in large clusters in dysplastic dentin-inducted areas (H&E stain, ×100).

The recurrent lesion found after 11 months, showed on grossing, a predominantly solid tumor mass (Figures 5(a) and 5(b)). Histopathological examination was in unison with the previous reports and findings. The only variation seen was large number of ghost cells forming islands and sheets and some were undergoing calcification (Figures 6(a) and 6(b)). These islands were seen getting incorporated into the surrounding lamellar/trabecular bone at few sites.

### 1.2. Case 2

**Figure 7:**
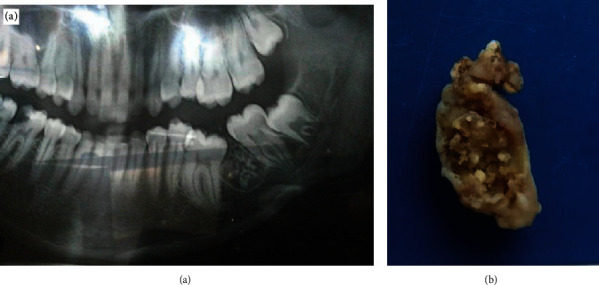
(a) OPG shows impacted 36 with a well-defined radiolucent lesion with specks of radioopacity in interradicular area of 36 and 37. (b) Grossing shows solid tissue with hard specks.

A 21-year-old female reported to the clinic with a submerged, partially erupted lower left molar (36). On clinical examination, the tooth was partially erupted and malaligned. No complaint of mobility or swelling was seen. On radiographic examination, a well-defined lesion was seen distal to 36. In between the roots of 36 and 37, a well-demarcated radiolucency with multiple specks of radiopacities was noted approximately 1 × 1.5 cm in size (Figure 7(a)). The gross specimen showed solid tissue with hard calcified specks (Figure 7(b)). The lesion was provisionally diagnosed as odontome and was treated with conservative excision to which the patient responded with complete healing.

**Figure 8:**
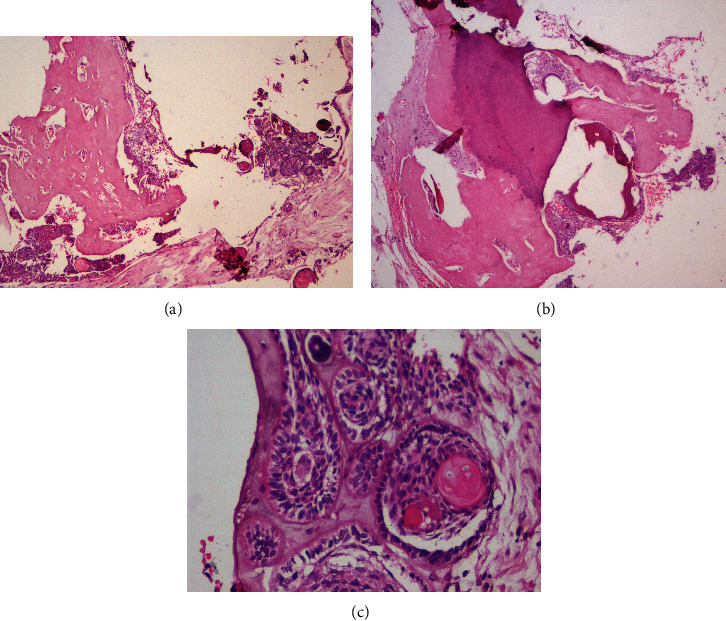
(a) Cystic lesion with epithelium showing ameloblastic change and large areas of dentinoid induction (H&E stain, ×40). (b) Dentin with predentin-like areas in the connective tissue (H&E stain, ×40). (c) Odontogenic epithelium is seen as small and large ameloblastic follicles with entrapped ghost cells (H&E stain, ×100).

Histopathological assessment of the H&E-stained sections revealed an encapsulated lesional cystic mass with epithelium showing ameloblastic changes and large ameloblastic follicles with entrapped ghost cells. Large areas of dentinoid induction and dentin with predentin-like areas in connective tissue were also evident (Figures 8(a)–8(c)). A histopathological diagnosis of DGCT was given. There were no complaints of recurrence after 3 years of follow-up.

## 2. Discussion

Calcifying odontogenic cyst (COC) is one of the mixed odontogenic lesions, which has traversed through a series of nomenclature and variations in pathology. In 1971, the World Health Organization (WHO) categorized COC as a “nonneoplastic cystic lesion” which ended in the emergence of a monistic and dualistic concept. The result culminated in 1992 with WHO naming the entity as neoplastic, irrespective of its cystic nature. In 2006, dentinogenic ghost cell tumor (DGCT) was the name given to solid variants as suggested by Praetorius et al. [1, 2, 5].

Thus, DGCT itself is considered a solid variant of COC. Though the tumor manifests either as a solid or a cystic lesion, it can present as a central tumor in the bone as an intraosseous type or can be a more contained tumor with a peripheral presence in the soft tissue [8].

Most commonly, DGCT presents as a central lesion in the 3^rd^ to 8^th^ decade of life, frequently in males [7]. It is a rare tumor with a prevalence of 0.3-0.5% of odontogenic tumors occurring in the posterior segment of the jawbones. Around 45 cases have been reported, and more than half have been observed in Asian patients [2, 6, 7, 9]. Among our cases, a deviation from the norm was seen as both the patients were in their early second decade, the tumor presenting in the posterior quadrant of the maxilla and mandible, respectively, as an intraosseous growth, one growing aggressively and the second comparatively a silent lesion.

Radiologically, DGCT appears as a unilocular or multilocular radiolucent and radiopaque lesion which is well demarcated [5, 9, 10]. The first case showed a large multilocular radiolucent lesion, partially well defined with radiopacity, an impacted canine, and a well-defined radiopaque mass in the mesial vicinity. The second case showed a classical unilocular well-defined radiolucent lesion with radiopacities in specks. The presentation was in unison with the cases of Patankar et al. [5], Bafna et al. [8], and Garcia et al. [9].

The surgical grossing revealed that the lesion was removed in toto in case 1 and was a cystic lesion with a hard odontome-like mass in close association. The case recurred as a completely solid tumor (Figures 5 and 6). Case 2 also presented as a well-circumscribed solid tumor with some grit.

The histopathology in the cases of DGCT shows the presence of an odontogenic epithelium in the form of cystic epithelium, islands, and nests. The odontogenic epithelium may exhibit tall columnar cells with reversal of polarity along with the presence of ghost cells. The mesenchymal component shows induction of dentinoid, and dentin-like tissue with ghost cells being a significant part of the tumor [9]. In the first case, the tumor showed the odontogenic epithelium in a cystic form with tall columnar basal cells with palisading nuclei. Suprabasilar cells resembled stellate reticulum-like cells along with ghost cells. The solid areas of the capsular wall showed the odontogenic epithelium arranged in islands of varying sizes with ghost cells. Numerous areas showed calcification of the ghost cell clusters. Masses of dentinoid with odontogenic cell inclusions were seen in close association of the odontogenic epithelium.

The ghost cells are said to be odontogenic epithelial cells with aberrant keratinization or coagulation necrosis. These cells are also seen in pilomatrixoma, odontoma, ameloblastic fibroma, and craniopharyngioma. In instances, they undergo calcifications as in our case. Dentin expressed can be a tubular structure similar to normal dentin or have an appearance of an eosinophilic irregular mass [1]. Our case showed the presence of the classic features of ghost cells along with recognizable dentin. Both intraosseous and peripheral variants of DGCT exhibit similar histopathological features, as observed in our case [10].

In case 1, the hard mass showed dentin in normal and dysplastic form, cementum-like areas, and enamel-like areas all arranged haphazardly along with ghost cells. The lesional mass resembled a composite complex odontome. In the same case, the recurrent tumor was completely solid and looked similar to the primary tumor.

In case 2, the tumor was well encapsulated and had a cystic element. The histopathology of which resembled case 1.

The variation in histopathology in our cases was the presence of ghost cells in close vicinity of the surrounding bone. A large number of ghost cell clusters were seen trapped in between the trabeculae of the bone.

Both the lesions were treated conservatively, but the first case recurred after 11 months. From the observations seen in the two cases, we cannot underestimate the behaviour of DGCT. The presentation though might be well contained or restricted. It can behave subtly, or actively as an aggressive tumor with high recurrence [1, 10, 11]. This calls for an aggressive line of treatment selection and free margins [5].

## 3. Conclusion

DGCT is a rare tumor that may behave aggressively with a recurrence rate of 33-73% [2, 10, 11] within a 1 to 20-year span. This stands true in our first case which not only recurred in less than 1 year as a 2.5 cm mass, but also switched over from a cystic tumor to a true solid tumor clarifying the not-so-benign nature of DGCT. This calls for a well-planned, suitable, aggressive surgical treatment ensuring clear margins. [5]. The second case did not show any recurrence owing to apt treatment and early diagnosis. Hence, highlighting the fact that some odontogenic tumors though called solid, can arise as cystic tumors. Appropriate treatment and management with follow-up is very fundamental in such cases. The aggressive nature and high recurrence rate make timely recall mandatory [5, 7]. The intraosseous variants appear to be more destructive than other forms [10, 9].

## Data Availability

The manuscript is a case series.
